# Assessment of atrioventricular interaction and response to balloon pulmonary angioplasty in chronic thromboembolic pulmonary hypertension using cardiac MRI

**DOI:** 10.1186/s13244-026-02453-8

**Published:** 2026-11-12

**Authors:** Hang Chen, Cheng Cheng, Yongxia Lei, Jiaxi Yu, Fahime Ghanbari, Qiuxi Lin, Xilai Chen, Chen Hong, Yu Deng

**Affiliations:** 1 Department of Radiology, The First Affiliated Hospital of Guangzhou Medical University (National Centre for Respiratory Medicine), No. 151 Yanjiang Road, Yuexiu District, Guangzhou, 510120 China; 2 Department of Radiology, Liwan Central Hospital, No. 289 Guangzhou Zhong Avenue, Liwan District, Guangzhou, 510170 China; 3 Department of Cardiovascular Medicine, Mayo Clinic, Rochester, MN 55905 USA; 4 Department of Pulmonary and Critical Care Medicine, The First Affiliated Hospital of Guangzhou Medical University, Guangzhou Institute of Respiratory Health, State Key Laboratory of Respiratory Disease, National Clinical Research Center for Respiratory Disease, Guangzhou, 510010 China

**Keywords:** Cine magnetic resonance imaging, Pulmonary hypertension, Myocardial contraction, Balloon angioplasty

## Abstract

**Objectives:**

This study aimed to evaluate atrioventricular interaction in patients with chronic thromboembolic pulmonary hypertension (CTEPH) and to determine the effects of balloon pulmonary angioplasty (BPA) on reversible cardiac remodeling assessed by cardiac magnetic resonance (CMR) imaging.

**Materials and methods:**

In this prospective single-center study, 52 patients with CTEPH (mean age: 58 ± 11 years; 34 females) and 35 healthy controls underwent CMR imaging. Twenty-five patients (mean age: 60 ± 12 years; 19 females) who underwent BPA received follow-up CMR at a mean interval of 7 ± 5 months. Cardiac structure, function, myocardial deformation, and epicardial adipose tissue (EAT) were assessed based on cine images.

**Results:**

Compared with controls, patients with CTEPH demonstrated predominant right-sided cardiac involvement, characterized by reduced right ventricular ejection fraction (*p* < 0.001), marked enlargement of right atrial (RA) and ventricular (RV) volumes, and severely impaired right heart strain (all *p* < 0.001). Left ventricular systolic function was relatively preserved, with mildly impaired global circumferential strain (*p* = 0.011) and a trend toward attenuated longitudinal strain (*p* = 0.055), whereas all left atrial (LA) strain components were markedly impaired (all *p* < 0.05), indicating altered atrioventricular interaction. Following BPA, the right heart structure, RV function and deformation, and LA conduit strain improved. EAT was comparable between groups and remained unchanged after BPA.

**Conclusion:**

CTEPH involves marked right heart systolic dysfunction and early biventricular diastolic abnormalities associated with impaired atrioventricular interaction. BPA could reverse cardiac remodeling by improving right heart structure, function, and partially recovering atrial deformation after pressure relief.

**Key Points:**

***Questions*** Atrioventricular interaction abnormalities and reversible cardiac remodeling in chronic thromboembolic pulmonary hypertension (CTEPH) after balloon pulmonary angioplasty (BPA) remain insufficiently characterized.

***Findings*** CTEPH is associated with impaired right heart strain and altered atrioventricular interaction; BPA improves right heart structure and function and partially recovers atrial deformation.

***Critical relevance statement*** Cardiac MRI-derived myocardial deformation and atrial phasic function may reflect alterations in atrioventricular interaction in CTEPH and complement conventional metrics in assessing cardiac remodeling and recovery after BPA.

**Graphical Abstract:**

**Figure:**
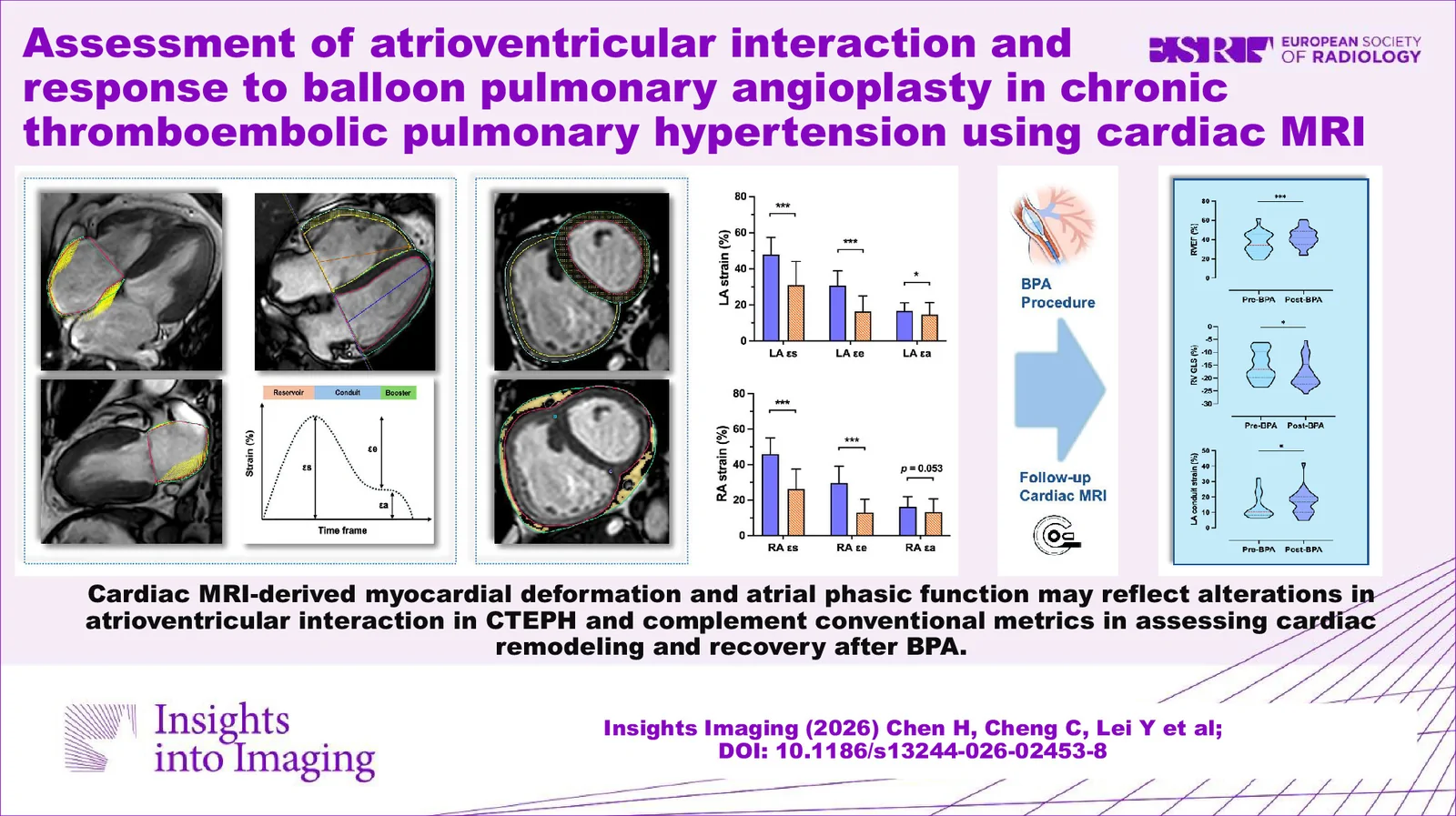

## Introduction

Chronic thromboembolic pulmonary hypertension (CTEPH) is a distinct subtype of pulmonary hypertension (PH), characterized by persistent obstruction of major pulmonary arteries by organized thrombi and microvascular arteriopathy in non-obstructed lung regions [1, 2]. The resulting elevation in pulmonary vascular resistance (PVR) leads to chronic right ventricular (RV) pressure overload and progressive right heart impairment. While assessments of RV structure and function are central in CTEPH, a sole ventricular parameter may fail to identify subtle perturbations in cardiac remodeling and atrioventricular interaction, particularly those related to early diastolic abnormalities.

In this context, atrial phasic mechanics, including reservoir, conduit, and booster pump functions, have been recognized as sensitive indicators of cardiac performance across cardiovascular diseases [3–5]. Because atrial function is closely correlated with ventricular relaxation, compliance and interventricular dependence, atrial phasic function analysis may provide sensitive insights into diastolic function, thereby complementing mechanistic information beyond conventional volumetric and systolic metrics in CTEPH.

Right heart catheterization (RHC) is the gold standard for hemodynamic assessment in PH, but it is invasive and fails to capture perivascular structures. Cardiac magnetic resonance (CMR) enables comprehensive, reproducible, and noninvasive assessment of cardiac structure and function and is increasingly essential in PH evaluation [6]. Building on advances in CMR, feature-tracking (FT)-CMR allows semi-automatic quantification of myocardial deformation from routine cine images. Although atrial strain has demonstrated prognostic relevance in PH [7–10], its role in atrioventricular coupling and remodeling in CTEPH remains unclear. Additionally, epicardial adipose tissue (EAT) is a metabolically active fat depot associated with inflammation and prognosis in PH [11]. As EAT may reflect alterations in the inflammatory and metabolic milieu accompanying cardiac remodeling, its characteristics and responsiveness to pressure unloading in CTEPH have not yet been elucidated.

Balloon pulmonary angioplasty (BPA) is an effective therapy for inoperable CTEPH and has been shown to achieve both hemodynamic and functional improvement [12]. Previous BPA studies in CTEPH have mainly concentrated on the effect on RV dysfunction [13], ventricular deformation [14], and cardiopulmonary hemodynamics [15]. Whether BPA also modulates atrioventricular coupling, atrial phasic function, and EAT, thereby contributing to reversible cardiac remodeling, remains unclear.

Therefore, the objectives of this study were to (1) comprehensively evaluate cardiac structure, function, and EAT in CTEPH patients; (2) quantify myocardial deformation using FT-CMR, with particular emphasis on atrial phasic strain as a surrogate marker of atrioventricular interaction and early diastolic abnormalities; and (3) investigate the effect of BPA on these cardiac alterations in order to characterize the extent of reversible cardiac remodeling.

## Materials and methods

### Study design and population

**Fig. 1:**
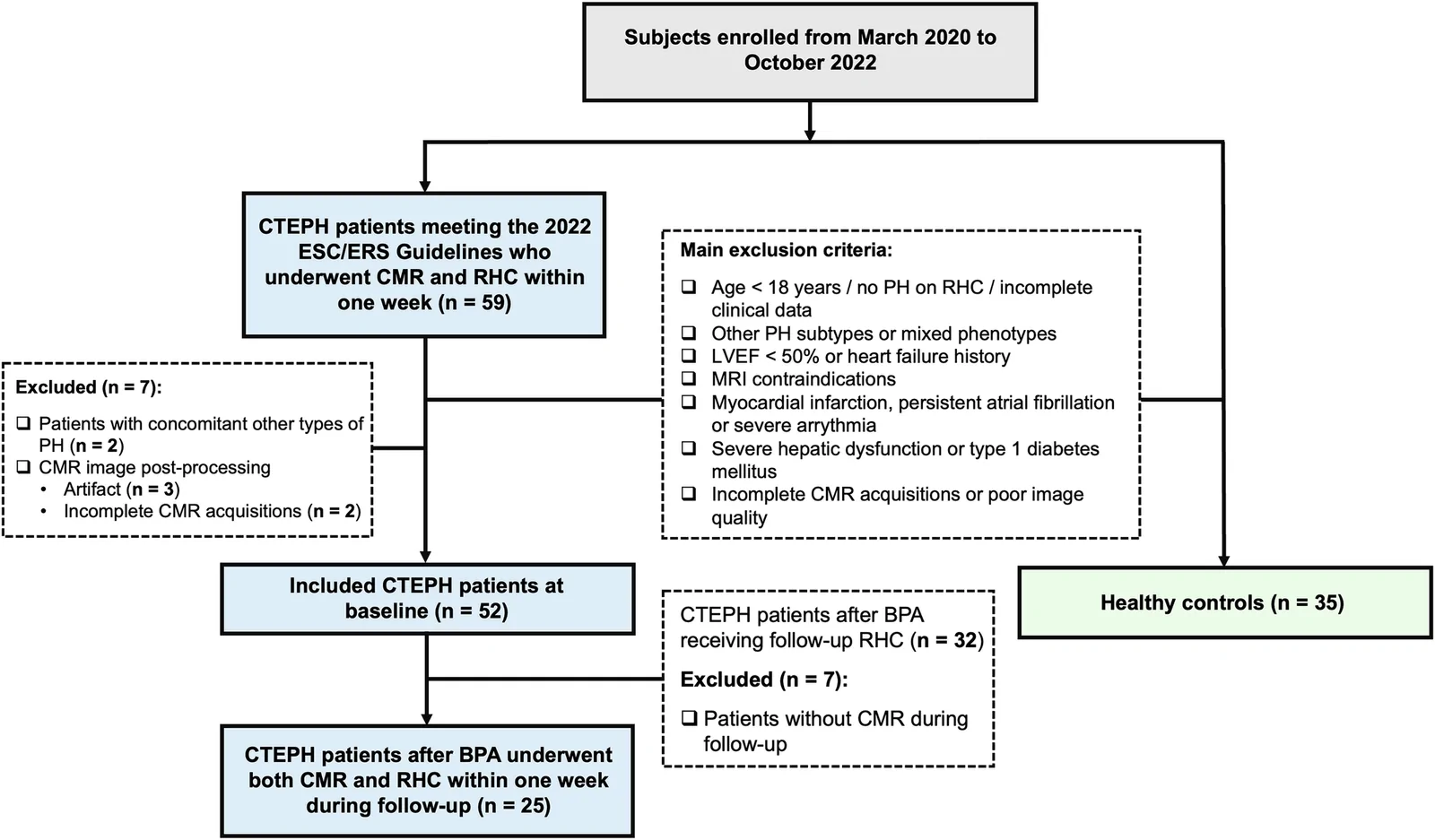
Study flowchart of enrolled cohorts and excluded subjects. BPA, balloon pulmonary angioplasty; CMR, cardiac magnetic resonance; CTEPH, chronic thromboembolic pulmonary hypertension; ESC, European Society of Cardiology; ERS, European Respiratory Society; PH, pulmonary hypertension; RHC, right heart catheterization

This prospective observational study aimed to evaluate cardiac structural and functional remodeling in patients with CTEPH using cine CMR. The primary outcome was the comprehensive assessment of cardiac structural and functional remodeling, with particular emphasis on atrial phasic function and atrioventricular interaction. The secondary outcome was the evaluation of changes in these CMR parameters following BPA. The study was approved by the local Ethics Committee of First Affiliated Hospital of Guangzhou Medical University (Ethics Approval No. [2020] 108) and conducted in accordance with the Declaration of Helsinki. Written informed consent was obtained from all participants. A consecutive cohort of patients with CTEPH was screened in this study between March 2020 and October 2022. According to the updated 2022 ESC/ERS Guidelines [2], the detailed definition and exclusion criteria for CTEPH are provided in the Supplemental Methods. Additionally, 35 healthy volunteers with no history of cardiovascular or systemic diseases were prospectively recruited as controls and underwent CMR for this study. All participants demonstrated normal CMR findings (see flowchart in Fig. 1).

### Hemodynamic and laboratory assessments

RHC examination was performed within 1 week (mean interval: 3 ± 2 days) following CMR to ensure temporal consistency between hemodynamic and imaging assessments. RHC metrics included mean pulmonary arterial pressure (mPAP), pulmonary artery wedge pressure (PAWP), PVR, cardiac output (CO) and cardiac index (CI) measured by Fick’s method. Patients underwent repeat RHC examination after BPA, and the interval to the follow-up CMR was consistently within 1 week. Blood samples were collected before CMR to measure creatine kinase (CK), creatine kinase muscle-brain fraction (CK-MB), high-sensitivity cardiac troponin I (hs-cTnI), and N-terminal pro-B-type natriuretic peptide (NT-proBNP).

### Follow-up protocol

After BPA, 32 patients with CTEPH were enrolled for follow-up assessment. Patients underwent one or more BPA sessions after baseline CMR, as required by treatment. Among them, seven patients did not undergo follow-up CMR and were therefore excluded from the longitudinal analysis. A total of 25 patients who underwent follow-up CMR before RHC (mean interval: 3 ± 2 days) were finally included to assess the therapeutic effect of BPA. The mean interval between the final BPA session and follow-up CMR was 7 ± 5 months (Fig. 1).

### Cardiac MR protocol

All CMR examinations were performed on a 3.0-T dual-source, dual-gradient MR scanner (Achieva TX or Ingenia CX; Philips Healthcare) equipped with a 16-channel SENSE XL-Torso phased-array surface coil. Respiratory and electrocardiographic gating were applied, and cine images were acquired using a steady-state free-precession (SSFP) sequence during end-expiratory breath-hold. Protocol details are available in the Supplemental Methods.

### Cardiac MR analysis

**Fig. 2:**
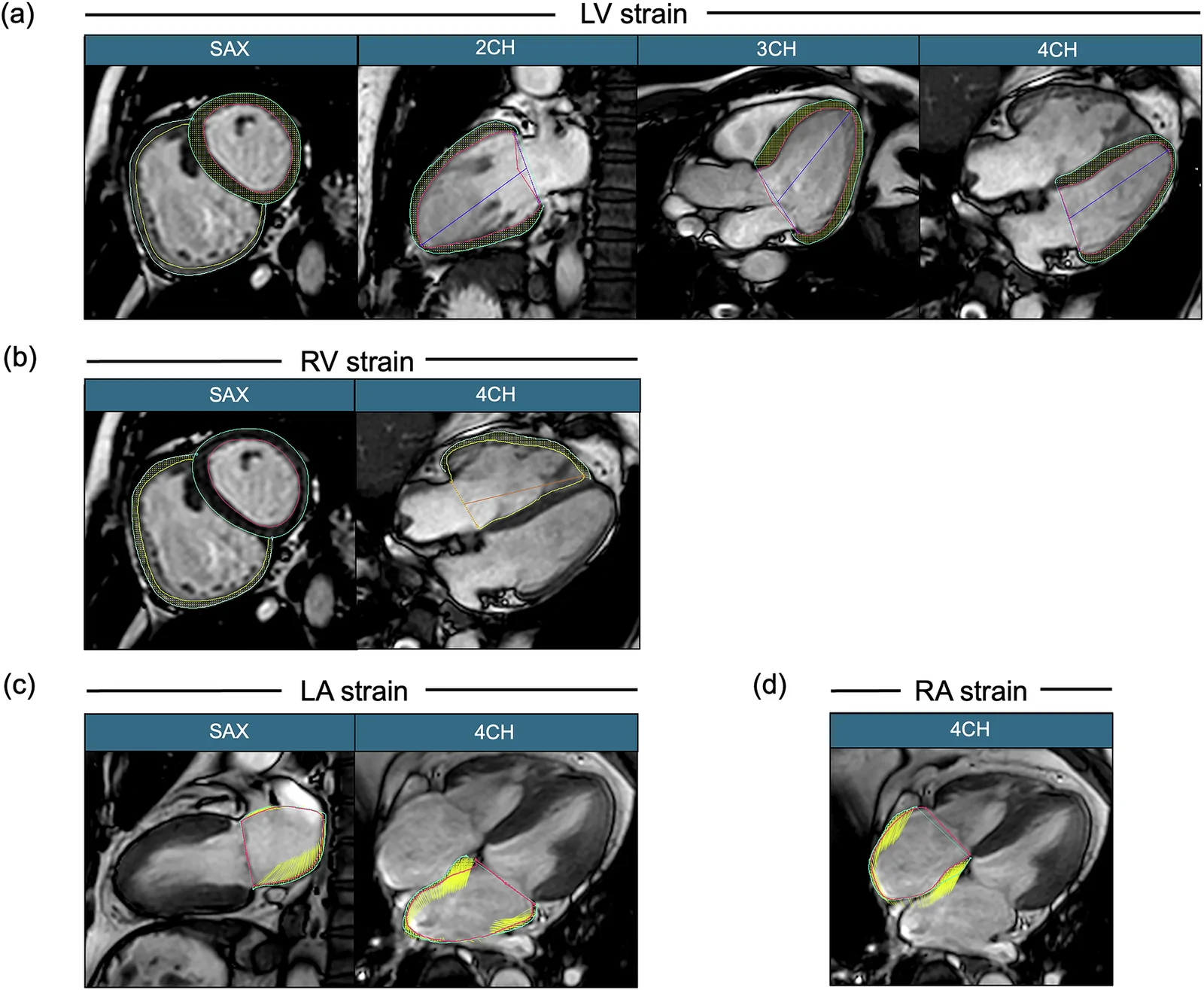
Representative examples of strain measurements using feature-tracking CMR imaging. Representative depiction of endocardial and epicardial boundaries and myocardial tracking points for the LV (SAX and three long-axis views) (**a**) and RV (SAX and 4-chamber views) (**b**), respectively. Representative plots illustrating LA (**c**) and RA (**d**) strain tracking at distinct phases of the cardiac cycle. 2CH, two-chamber view; 3CH, 3-chamber view; 4CH, 4-chamber view; LA, left atrial; LV, left ventricular; RA, right atrial; RV, right ventricular; SAX, short-axis view

Two independent investigators (H.C. and C.C.), with 5 and 3 years of CMR experience, respectively, blinded to clinical data, analyzed each CMR randomly using cvi42^®^ software (version 6.1.3). CMR-derived parameters were reported as the mean values of two investigators. According to the Society for Cardiovascular Magnetic Resonance (SCMR) Guidelines [16], conventional cardiac parameters, including end-diastolic volume (EDV), end-systolic volume (ESV), stroke volume (SV), ejection fraction (EF), and LV mass, were obtained using a semi-automated contouring approach with manual adjustment of endocardial and epicardial borders at end-systole and end-diastole across a full stack of SAX cine images. Left atrial (LA) volumes were calculated using the biplane area-length method, and right atrial (RA) volumes were calculated using the monoplane method, excluding pulmonary veins and atrial appendages. Ventricular global longitudinal, circumferential, and radial strain, as well as atrial phasic strain, were quantified using a tissue-tracking module on cine images (Fig. 2), following previously described and validated protocols [5, 17–21]. Detailed contouring and calculations are provided in the Supplemental Methods.

### Epicardial adipose tissue quantification

EAT volume and mass were quantified by manually tracing the epicardial contours of the ventricles from the mitral annulus to the apex at end-diastole on SAX cine images (Supplemental Fig. S2). The epicardium and visceral pericardium were delineated using the LV analysis tool on each slice, and intravascular lumina within the traced region were manually excluded [22].

### Intra-observer and inter-observer reproducibility

Intra- and inter-observer reproducibility were evaluated in 15 randomly selected patients (28.8%). Strain and EAT measurements were repeated by the same investigator after an interval of at least 1 week and independently analyzed by a second investigator.

### Statistical analysis

The normality of continuous variables was assessed using the Kolmogorov–Smirnov test. Normally distributed variables are presented as mean ± standard deviation and were compared using the Student’s *t*-test. Non-normally distributed variables are presented as medians (Q1–Q3) and were compared using the Mann–Whitney *U*-test or the Wilcoxon matched-pairs signed-rank test. Categorical variables were summarized as counts and percentages and compared using the Chi-square or Fisher's exact test, as appropriate. Inter- and intra-observer reproducibility of strain and EAT measurements was assessed using the intraclass correlation coefficient (ICC). Pearson’s or Spearman’s correlation coefficients (*r*) were calculated as appropriate. Scatter plots with fitted linear regression lines were generated for visualization. Multivariable linear regression analyses were performed to evaluate whether interaction effects influenced the associations between patient characteristics and atrial strain measurements. A two-sided *p*-value < 0.05 was considered statistically significant. All analyses and figure plotting were performed using SPSS Statistics (version 31), GraphPad Prism (version 10.6.0), and RStudio (version 2022.07.2) on macOS.

## Results

### Demographics and clinical characteristics

A total of 52 CTEPH patients were finally included. Demographics and clinical characteristics are summarized in Table 1. Based on hemodynamics and the updated 2022 ESC/ERS Guidelines [2], 12 patients (23.1%) were classified as mild PH, 28 (53.8%) as moderate PH, and 12 (21.1%) as severe PH. Baseline characteristics of patients with and without follow-up CMR are provided in Supplemental Table S1.

**Table 1:** Demographics and clinical characteristics of healthy controls and CTEPH patients at baseline and after BPA

	Controls (*n* = 35)	All CTEPH Baseline (*n* = 52)	*p-*value^a^	CTEPH before BPA Baseline (*n* = 25)	CTEPH after BPA Follow-up (*n* = 25)	*p-*value^b^
Demographics						
Age, years	40 ± 15	58 ± 11	**< 0.001**	60 ± 12	60 ± 12	-
Female, *n* (%)	21 (60)	34 (65.4)	0.776	19 (76)	19 (76)	-
Height, cm	163 ± 8	161 ± 8	0.372	159 ± 8	158 ± 9	0.253
Weight, kg	62 ± 11	62 ± 10	0.944	60 ± 8	61 ± 9	0.225
Body surface area, m²	1.7 ± 0.2	1.7 ± 0.2	0.894	1.6 ± 0.1	1.6 ± 0.1	0.547
BMI, kg/m²	23 ± 4	24 ± 4	0.441	24 ± 3	24 ± 3	0.127
Clinical parameters						
6MWD, m	-	424 ± 92		390 ± 92	440 ± 93	**0.009**
WHO class, *n* (%)						**0.034**
I	-	5 (12.5)		1 (4.2)	2 (9.1)	
II	-	25 (62.5)		14 (58.3)	16 (72.7)	
III	-	8 (20)		8 (33.3)	3 (13.6)	
IV	-	2 (5)		1 (4.2)	1 (4.5)	
Number of BPA sessions	-	-		-	2 ± 1	-
Blood biomarkers						
NT-proBNP, pg/mL	-	234 (78, 760)		594 (157, 1056)	94 (55, 253)	**0.003**
hs-cTnI, pg/mL	-	5.8 (3.4, 10.8)		7.8 (5.2, 10.5)	4.8 (3, 10)	**0.005**
CK, U/L	-	82 ± 41		78 ± 38	90 ± 58	0.061
CK-MB, U/L	-	11 (9, 14)		10 (9, 12.8)	10 (8.5, 13.5)	0.423
RHC metrics						
mPAP, mmHg	-	40 ± 12		44 ± 11	36 ± 9	**0.001**
SVCP, mmHg	-	6 ± 3		6 ± 3	7 ± 3	0.139
IVCP, mmHg	-	6 ± 3		6 ± 3	7 ± 3	0.185
RAP, mmHg	-	6 ± 3		6 ± 3	7 ± 3	0.109
PAWP, mmHg	-	9 ± 3		9 ± 3	8 ± 3	0.712
PVR, WU	-	7 ± 4		8 ± 4	6 ± 2	**0.018**
CO^c^, L/min	-	5.2 ± 1.7		5.0 ± 2.0	4.9 ± 0.9	0.888
CI^c^, L/min/m²	-	3.2 ± 1.1		3.2 ± 1.4	3.0 ± 0.6	0.631
PH severity^d^, *n* (%)						**0.013**
Mild	-	12 (23.1)		3 (12)	5 (20)	
Moderate	-	28 (53.8)		14 (56)	17 (68)	
Severe	-	12 (21.1)		8 (32)	2 (8)	

Values are presented as mean ± SD or median (Q1, Q3) for continuous, and *n* (%) for categorical data. Significant *p*-values are shown in bold

^a^ *p*-value for all CTEPH patients vs. controls

^b^ *p*-value for CTEPH patients before BPA vs. after BPA

^c^ CO and CI were measured by Fick’s method

^d^ PH severity was defined as mild (20 mmHg < mPAP < 30 mmHg), moderate (30 mmHg ≤ mPAP < 50 mmHg), and severe (mPAP ≥ 50 mmHg) according to the 2022 ESC/ERS Guidelines [2]. One of 25 patients achieved normal mPAP (14 mmHg) after BPA

*6MWD* 6-min walk distance, *BMI* body mass index, *BPA* balloon pulmonary angioplasty, *CI* cardiac index, *CO* cardiac output, *CK-MB* creatine kinase muscle-brain fraction, *CTEPH* chronic thromboembolic pulmonary hypertension, *hs-cTnI* high-sensitivity cardiac troponin I, *IVCP* inferior vena cava pressure, *mPAP* mean pulmonary arterial pressure, *NT-proBNP* N-terminal pro-B-type natriuretic peptide, *PAWP* pulmonary artery wedge pressure, *PH* pulmonary hypertension, *PVR* pulmonary vascular resistance, *RAP* right atrial pressure, *RHC* right heart catheterization, *SVCP* superior vena cava pressure, *WU* wood units

### Clinical and hemodynamic changes after BPA

**Fig. 3:**
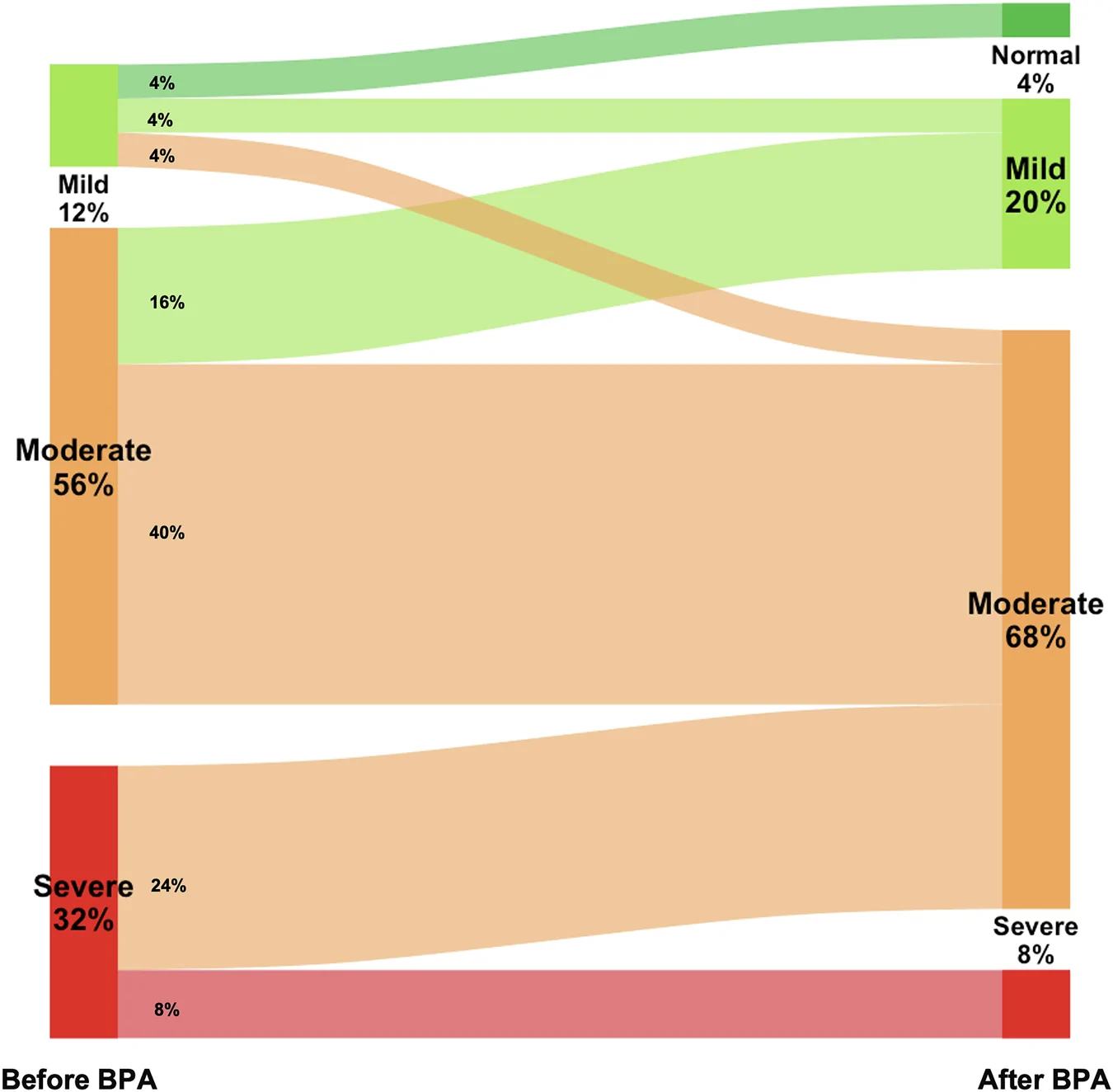
Sankey plot for the changes in PH severity. Bars represent the proportion of patients in different PH severity groups. Colors indicate the distribution before and after BPA. Abbreviations as in Fig. 1

Functional parameters recovered significantly after BPA, with improvements in the 6-WMD (*p* = 0.009) and the WHO functional class (*p* = 0.034) (Table 1). NT-proBNP (*p* = 0.003) and hs-cTnI (*p* = 0.005) levels decreased following BPA. Hemodynamic metrics also improved significantly, with reductions in mPAP (*p* = 0.001) and PVR (*p* = 0.018). Following BPA, the proportion of patients with severe PH decreased, accompanied by a corresponding increase in those classified as having mild or moderate PH (*p* = 0.013) (Fig. 3).

### CMR parameters in CTEPH vs. controls

Intra- and inter-observer reproducibility was good to excellent for strain and EAT measurements (Supplemental Table S2). CMR parameters for both the CTEPH patients and healthy controls are summarized in Table 2. Regarding left heart function, CTEPH patients showed lower LVEF (60 ± 7% vs. 63 ± 5%; *p* = 0.024) and LAEF (57 ± 12% vs. 62 ± 7%; *p* = 0.047) compared with controls. In addition, CTEPH patients showed a reduced LVSVi (*p* = 0.024) and an increased LAEDVi (*p* < 0.01). Right heart assessment revealed more pronounced functional impairments in CTEPH, both RVEF (39 ± 11% vs 53 ± 6%; *p* < 0.001) and RAEF (44 ± 12% vs. 54 ± 8%; *p* < 0.001) in CTEPH were significantly reduced. Right atrial and ventricular end-diastolic and end-systolic volumes were significantly increased (all *p* < 0.001).

**Table 2:** CMR anatomical, functional and strain parameters of healthy controls and all CTEPH patients at baseline

	Controls (*n* = 35)	All CTEPH baseline (*n* = 52)	*p-*value
CMR—left heart			
Heart rate, beats/min	70 ± 8	73 ± 12	0.292
LV cardiac index, L/min/m^2^	3.1 ± 0.6	2.8 ± 0.7	0.053
LVEF, %	63 ± 5	60 ± 7	**0.024**
LV mass index, g/m²	44 ± 7	44 ± 7	0.799
LVEDVi, mL/m²	71 ± 12	65 ± 16	0.064
LVESVi, mL/m²	27 ± 7	26 ± 7	0.695
LVSVi, mL/m²	45 ± 7	39 ± 12	**0.024**
LAEDVi, mL/m²	12 ± 3	16 ± 10	**< 0.01**
LAESVi, mL/m²	27 ± 8	31 ± 14	0.219
LAEF, %	62 ± 7	57 ± 12	**0.047**
CMR—right heart			
RV cardiac index, L/min/m^2^	2.7 ± 0.5	2.8 ± 0.9	0.795
RVEF, %	53 ± 6	39 ± 11	**< 0.001**
RVEDVi, mL/m²	73 ± 12	104 ± 38	**< 0.001**
RVESVi, mL/m²	34 ± 8	65 ± 32	**< 0.001**
RVSVi, mL/m²	39 ± 7	39 ± 13	0.532
RAEDVi, mL/m²	13 ± 5	30 ± 30	**< 0.001**
RAESVi, mL/m²	24 ± 7	45 ± 34	**< 0.001**
RAEF, %	54 ± 8	44 ± 12	**< 0.001**
CMR—strain			
LV GLS, %	−15 ± 3	−13 ± 4	0.055
LV GCS, %	−21 ± 3	−19 ± 3	**0.011**
LV GRS, %	43 ± 11	39 ± 11	0.160
RV GLS, %	−23 ± 5	−16 ± 7	**< 0.001**
RV GCS, %	−14 ± 3	−11 ± 4	**< 0.001**
RV GRS, %	25 ± 6	17 ± 7	**< 0.001**
LA total strain (εs), %	48 ± 9	31 ± 13	**< 0.001**
LA conduit strain (εe), %	31 ± 8	17 ± 8	**< 0.001**
LA booster strain (εa), %	17 ± 4	15 ± 7	**0.046**
RA total strain (εs), %	46 ± 9	26 ± 11	**< 0.001**
RA conduit strain (εe), %	30 ± 9	13 ± 7	**< 0.001**
RA booster strain (εa), %	16 ± 6	13 ± 7	0.053
CMR—epicardial adipose tissue			
EAT volume, mL	41 ± 17	44 ± 17	0.561
EAT mass, g	43 ± 17	46 ± 18	0.573
EAT volume index, mL/m²	24 ± 9	26 ± 10	0.483
EAT mass index, g/m²	25 ± 9	27 ± 10	0.492

Values are presented as mean ± SD. Significant *p*-values are shown in bold

*CMR* cardiac magnetic resonance, *CTEPH* chronic thromboembolic pulmonary hypertension, *EAT* epicardial adipose tissue, *EF* ejection fraction, *GCS* global circumferential strain, *GLS* global longitudinal strain, *GRS* global radial strain, *LA* left atrial, *LAEDVi* left atrial end-diastolic volume index, *LAESVi* left atrial end-systolic volume index, *LV* left ventricular, *LVEDVi* left ventricular end-diastolic volume index, *LVESVi* left ventricular end-systolic volume index, *RA* right atrial, *RAEDV*i right atrial end-diastolic volume index, *RAESV*i right atrial end-systolic volume index, *RV* right ventricular, *RVEDVi* right ventricular end-diastolic volume index, *RVESVi* right ventricular end-systolic volume index

**Fig. 4:**
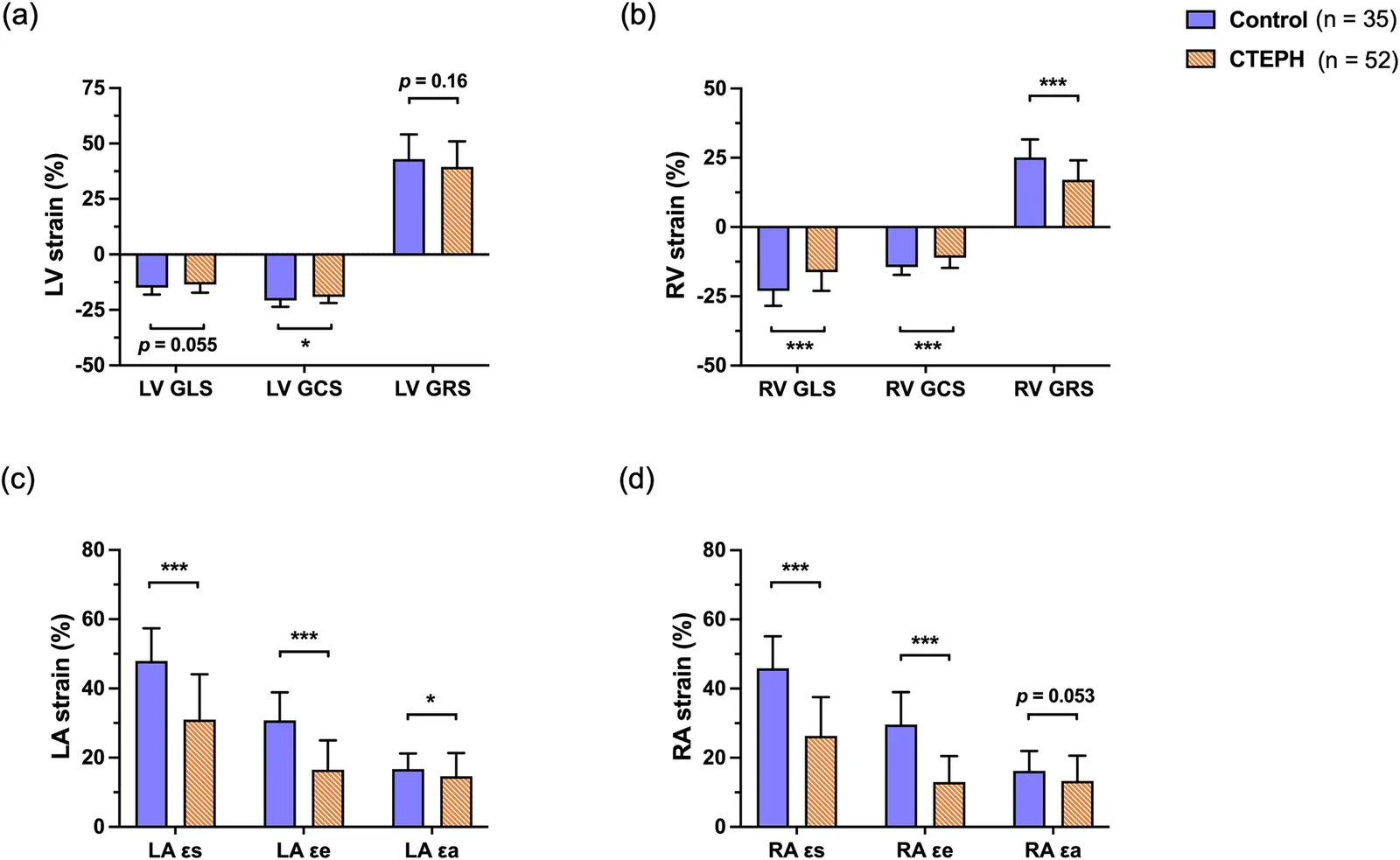
Comparisons of myocardial strain parameters between CTEPH patients and healthy controls. *p*-values for the unpaired *t*-test: *p* < 0.05 (*), *p* < 0.01 (**), *p* < 0.001 (***). CTEPH patients demonstrated (**a**) reduced LV GCS and a trend toward lower GLS, with preserved GRS. All RV (**b**), LA (**c**), and RA (**d**) strain parameters were markedly reduced, except for the RA booster strain, showing a downward trend. εs, total strain; εe, conduit strain; εa, booster strain; CTEPH, chronic thromboembolic pulmonary hypertension; LA, left atrial; LV, left ventricular; GCS, global circumferential strain; GLS, global longitudinal strain; GRS, global radial strain; RA, right atrial; RV, right ventricular

Table 2 and Fig. 4 summarize myocardial strain parameters in healthy controls and CTEPH patients. CTEPH demonstrated attenuated LV GCS (−19 ± 3% vs. −21 ± 3%; *p* = 0.011) and a trend toward reduced GLS (−13 ± 4% vs. −15 ± 3%; *p* = 0.055) compared with healthy controls, whereas LV GRS was not significantly distinct (*p* = 0.16). All RV strain indices were impaired in CTEPH (all *p* < 0.001). Both LA total (31 ± 13% vs. 48 ± 9%; *p* < 0.001) and conduit strain (17 ± 8% vs. 31 ± 8%; *p* < 0.001) were diminished, accompanied by a decline in booster strain (15 ± 7% vs. 17 ± 4%; *p* = 0.046). Similarly, RA total (26 ± 11% vs. 46 ± 9%; *p* < 0.001) and conduit strain (13 ± 7% vs. 30 ± 9%; *p* < 0.001) were markedly attenuated, with a trend toward lower booster strain (13 ± 7% vs. 16 ± 6%; *p* = 0.053) in CTEPH. Total EAT volume (*p* = 0.483) and mass index (*p* = 0.492) did not differ statistically between CTEPH patients and the healthy control group (Table 2).

Multivariable regression analysis showed no independent associations for LA strain parameters, whereas selected demographic and hemodynamic variables were independently associated with RA and ventricular strain parameters (Supplemental Tables S3–S6).

### CMR parameters in CTEPH pre-BPA vs. post-BPA

**Fig. 5:**
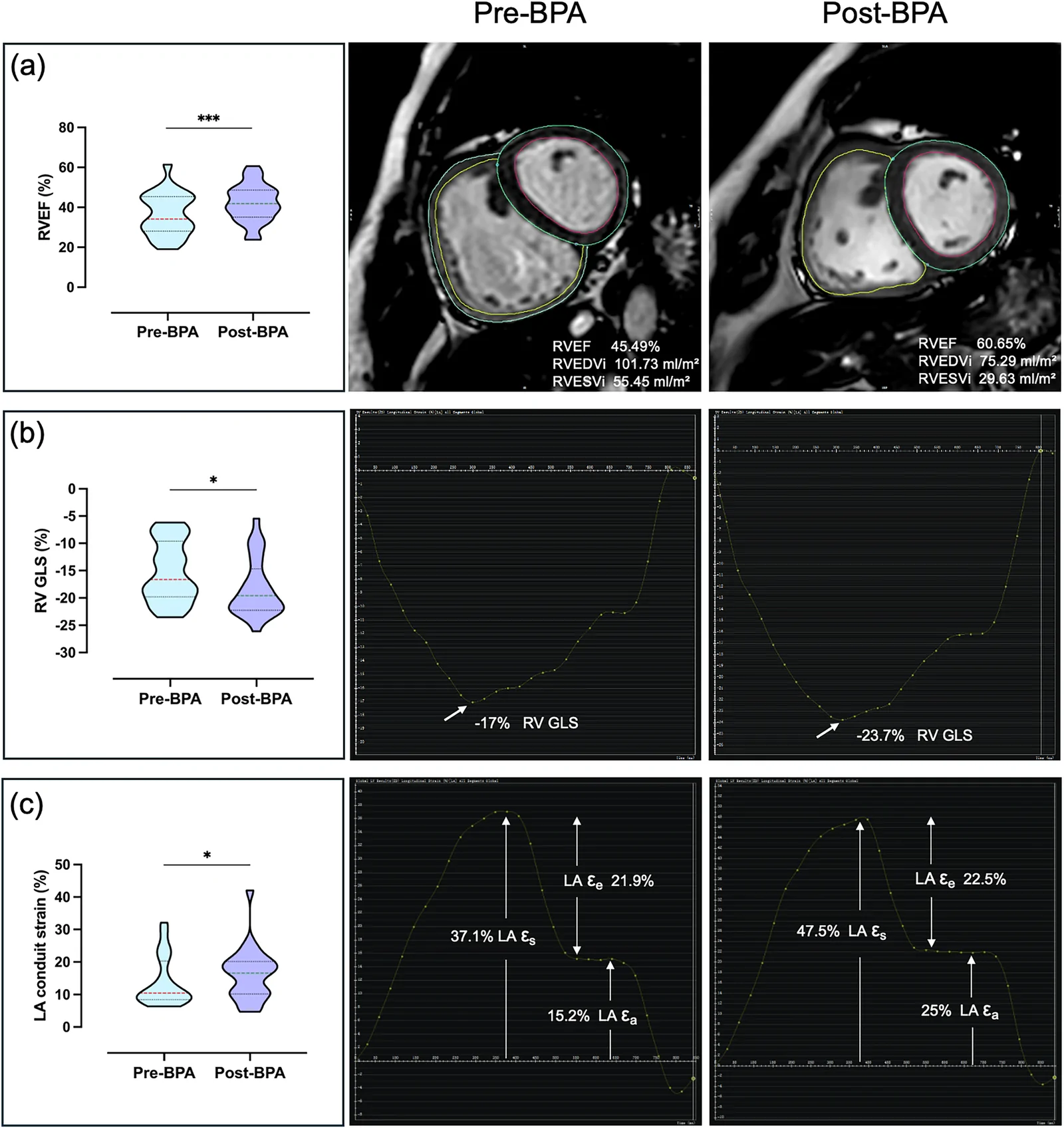
Changes of CMR-derived RVEF (**a**), RV GLS (**b**), and LA conduit strain (**c**) measurements pre- and post-BPA. *p*-values for paired *t*-test: *p* < 0.05 (*), *p* < 0.01 (**), *p* < 0.001 (***). The two panels on the right illustrate the alterations in RVEF and morphology (top), RV GLS (middle), and LA strain (bottom) before and after BPA in a 62-year-old female CTEPH patient. εs, total strain; εe, conduit strain; εa, booster strain; BPA, balloon pulmonary angioplasty; GLS, global longitudinal strain; LA, left atrial; RV, right ventricular; RVEDVi, right ventricular end-diastolic volume index; RVEF, right ventricular ejection fraction; RVESVi, right ventricular end-systolic volume index

RVEF improved significantly after BPA procedure (36 ± 11% to 42 ± 9%; *p* = 0.002; Fig. 5a, Table 3), accompanied by reductions in RVEDVi (114 ± 37 mL/m² vs. 95 ± 39 mL/m²; *p* < 0.001) and RVESVi (74 ± 32 mL/m² vs. 58 ± 33 mL/m², *p* < 0.001). LV metrics remained unchanged after BPA (all *p* > 0.05). LAEDVi increased (17 ± 13 mL/m² vs. 19 ± 11 mL/m²; *p* = 0.039) and RAESVi decreased (45 ± 30 mL/m² vs. 39 ± 25 mL/m²; *p* = 0.038) following BPA. Strain analysis showed significant improvements in RV GRS (15 ± 7% vs. 18 ± 8%; *p* = 0.049) and GLS (−15 ± 6% vs. −18 ± 5%; *p* = 0.012; Fig. 5b, Table 3), as well as increased LA conduit strain (14 ± 8% vs. 17 ± 8%; *p* = 0.038; Fig. 5c, Table 3), and RV GCS showed a trend toward improvement (*p* = 0.053) following BPA. No statistical changes were observed in EAT (*p* > 0.05).

**Table 3:** CMR parameters of CTEPH patients before and after balloon pulmonary angioplasty (BPA)

	CTEPH before BPA (*n* = 25)	CTEPH after BPA (*n* = 25)	*p-v*alue
CMR—left heart			
Heart rate, beats/min	76 ± 15	70 ± 12	**0.025**
LV cardiac index, L/min/m^2^	2.8 ± 0.8	2.8 ± 0.5	0.974
LVEF, %	59 ± 8	60 ± 6	0.434
LV mass index, g/m²	44 ± 8	45 ± 8	0.228
LVEDVi, mL/m²	63 ± 18	68 ± 14	0.115
LVESVi, mL/m²	26 ± 8	27 ± 7	0.107
LVSVi, mL/m²	38 ± 13	41 ± 9	0.205
LAEDVi, mL/m²	17 ± 13	19 ± 11	**0.039**
LAESVi, mL/m²	32 ± 18	34 ± 13	0.432
LAEF, %	54 ± 14	52 ± 10	0.468
CMR—right heart			
RV cardiac index, L/min/m^2^	3.0 ± 1.0	2.6 ± 0.6	0.058
RVEF, %	36 ± 11	42 ± 9	**0.002**
RVEDVi, mL/m²	114 ± 37	95 ± 39	**< 0.001**
RVESVi, mL/m²	74 ± 32	58 ± 33	**< 0.001**
RVSVi, mL/m²	39 ± 13	38 ± 9	0.370
RAEDVi, mL/m²	29 ± 24	26 ± 22	0.151
RAESVi, mL/m²	45 ± 30	39 ± 25	**0.038**
RAEF, %	44 ± 12	46 ± 11	0.552
CMR—strain			
LV GLS, %	−13 ± 4	−14 ± 4	0.339
LV GCS, %	−19 ± 3	−18 ± 3	0.171
LV GRS, %	38 ± 10	39 ± 9	0.633
RV GLS, %	−15 ± 6	−18 ± 5	**0.012**
RV GCS, %	−10 ± 4	−12 ± 4	0.053
RV GRS, %	15 ± 7	18 ± 8	**0.049**
LA total strain (εs), %	28 ± 11	31 ± 11	0.129
LA conduit strain (εe), %	14 ± 8	17 ± 8	**0.038**
LA booster strain (εa), %	15 ± 7	15 ± 5	0.797
RA total strain (εs), %	26 ± 11	27 ± 13	0.552
RA conduit strain (εe), %	11 ± 6	12 ± 9	0.726
RA booster strain (εa), %	15 ± 8	15 ± 8	0.621
CMR—epicardial adipose tissue			
EAT volume, mL	44 ± 18	41 ± 20	0.127
EAT mass, g	46 ± 19	44 ± 21	0.139
EAT volume index, mL/m²	27 ± 11	25 ± 11	0.079
EAT mass index, g/m²	28 ± 11	27 ± 12	0.087

Values are presented as mean ± SD. Significant *p*-values are shown in bold

*BPA* balloon pulmonary angioplasty, *CMR* cardiac magnetic resonance, *CTEPH* chronic thromboembolic pulmonary hypertension, *EAT* epicardial adipose tissue, *EF* ejection fraction, *GCS* global circumferential strain, *GLS* global longitudinal strain, *GRS* global radial strain, *LA* left atrial, *LAEDVi* left atrial end-diastolic volume index, *LAESVi* left atrial end-systolic volume index, *LV* left ventricular, *LVEDVi* left ventricular end-diastolic volume index, *LVESVi* left ventricular end-systolic volume index, *RA* right atrial, *RAEDVi* right atrial end-diastolic volume index, *RAESVi* right atrial end-systolic volume index, *RV* right ventricular, *RVEDVi* right ventricular end-diastolic volume index, *RVESVi* right ventricular end-systolic volume index

### Correlation of ΔNT-proBNP, ΔPVR with cardiac function and strain

**Fig. 6:**
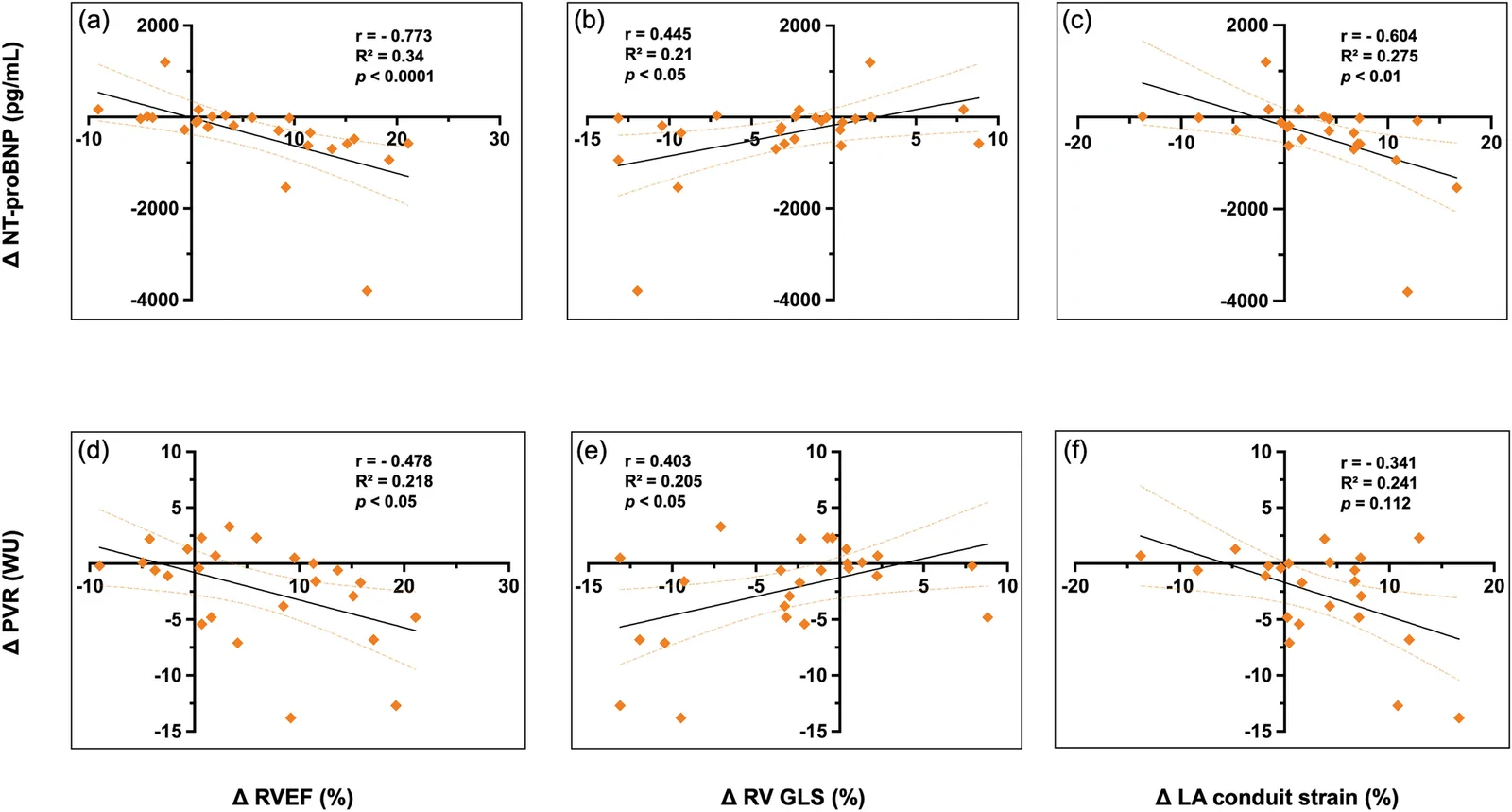
Correlations between changes in NT-proBNP, PVR, and cardiac functional and strain parameters. The plots illustrate that the decrease in NT-proBNP was associated with improved RVEF (**a**), RV GLS (**b**), and LA conduit strain (**c**), while the reduction in PVR correlated with improved RVEF (**d**) and RV GLS (**e**) but not LA conduit strain (**f**). GLS, global longitudinal strain; LA, left atrial; NT-proBNP, N-terminal pro-B-type natriuretic peptide; PVR, pulmonary vascular resistance; RV, right ventricular; RVEF, right ventricular ejection fraction

Figure 6 illustrates that the decrease in NT-proBNP was highly correlated with increases in RVEF (*r* = −0.773; *p* < 0.0001) and LA conduit strain (*r* = −0.604; *p* < 0.01). Also, the reduction in NT-proBNP correlated with improvement in RV GLS (*r* = 0.445; *p* < 0.05), where the negative sign indicates the direction of myocardial contractility. The decline in PVR was correlated with improved RVEF (*r* = −0.478; *p* < 0.05) and RV GLS (*r* = 0.403; *p* = 0.045), but not with changes in LA conduit strain (*r* = −0.341; *p* = 0.112).

## Discussion

This prospective study comprehensively assessed cardiac structure, function, myocardial deformation, and EAT in CTEPH patients using CMR imaging, with particular emphasis on atrial phasic strain as a surrogate marker of atrioventricular coupling. Furthermore, we evaluated the therapeutic effect of BPA. The key findings are as follows:

(1) CTEPH was characterized by predominant right-sided cardiac involvement, with impaired RV systolic function and marked chamber enlargement, while LV systolic involvement was relatively limited; (2) myocardial strain analysis demonstrated marked impairment of right heart strain and significant attenuation in LA strain. The impaired atrial strain indicates elevated LA pressure and early diastolic abnormalities secondary to RV pressure overload; (3) in the cohort undergoing BPA procedure, follow-up CMR demonstrated improvements in right heart structure, function, and partial recovery of atrial deformation following pressure relief; (4) EAT did not differ between CTEPH and controls, and remained unchanged after BPA.

Consistent with prior literature [23, 24], markedly elevated pulmonary arterial pressure and PVR lead to chronic RV pressure overload, chamber dilation, and subsequent RV dysfunction in CTEPH. By contrast, LV systolic function was relatively spared, as the primary hemodynamic disturbance is confined to the pulmonary circulation [25]. In agreement with Vos et al [26], although LVEF was slightly reduced in CTEPH due to RV-LV interaction, it remained within the normal range. This might be explained by the fact that reduced LV filling may result from ventricular interdependence and septal displacement secondary to RV dilation, while intrinsic LV systolic function is generally preserved [27]. These findings indicate that CTEPH predominantly involves right-sided cardiac remodeling due to pulmonary vascular obstruction, thereby distinguishing its pathophysiologic profile from left-heart driven PH [28].

All RV strain components were impaired in CTEPH, in agreement with previous studies [29, 30]. This is not unexpected, given that myocardial strain is a sensitive marker of subclinical myocardial dysfunction and is therefore reduced in the setting of RV dysfunction. However, only LV GCS showed a decline among LV strain measurements, suggesting that it may represent a more sensitive indicator of subclinical LV systolic involvement. This further supports the notion that LV strain predominantly represents myocardial shortening during systole and may be less sensitive to diastolic abnormalities [26].

Attenuated LA strain, particularly total and conduit strain, was observed in CTEPH patients compared with controls, similar to findings by Kishiki et al [9], who reported impaired LA total (reservoir) and booster functions in PH despite normal LVEF. During diastole, the pulmonary circulation, LA and LV are functionally coupled, and LA atrial strain has been proposed as a sensitive indicator of LV filling pressure associated with diastolic abnormalities [31, 32]. The reduced LA total and conduit strain indicate diminished LA expansion from pulmonary venous return and reduced passive LV filling. In CTEPH, RV pressure overload and dilation with interventricular septal shift may restrict LV diastolic filling and pulmonary venous return, thereby lowering LV preload [26]. Accordingly, impaired LA strain is probably attributable to atrioventricular coupling dysfunction and reduced LV preload [9].

Using echocardiography, Roca et al [33] demonstrated marked RA dilation with impaired RA reservoir and conduit functions but largely preserved booster function in PH. The chronic elevation of RV afterload increases filling pressures that are transmitted backward to the RA, resulting in progressive RA expansion to maintain right-sided CO [23]. Sustained wall stress and myocardial stretch subsequently reduce atrial compliance and contractility, leading to the deterioration of RA reservoir and conduit functions [7], as evidenced by the impaired RA strain in this study. Taken together, relatively preserved RA booster function likely represents a compensatory mechanism to persist atrial emptying when reservoir and passive filling functions decline, indicating ongoing atrial remodeling and mechanical dysfunction [33].

No significant difference in EAT was observed between CTEPH and controls, in contrast to Chen et al [11], who reported reduced EAT in a heterogeneous PH population. This discrepancy might reflect the unique pathophysiology of CTEPH, which primarily involves chronic thromboembolic obstruction and pulmonary artery remodeling. Given that EAT is closely associated with metabolic syndrome and coronary artery disease, and reflects systemic metabolic and inflammatory status [22, 34]. Thus, EAT changes may be less pronounced in CTEPH than in other PH subtypes.

As expected and similar to prior studies [35], BPA significantly improved hemodynamics and functional parameters, including reductions in mPAP, PVR, NT-proBNP, and RV volumes, along with improved RV systolic function as indicated by increased RVEF and myocardial deformation. RVEF represents overall RV systolic function by integrating both longitudinal and radial mechanics [35]. In line with Kawakubo et al [14], BPA significantly improved RV GLS and GRS, while RV GCS showed only a nonsignificant upward trend. A possible explanation lies in the structural characteristics of the RV, which consists of two myocardial fiber layers and primarily deforms along the longitudinal axis through both contraction and torsion [36, 37]. Since RV GCS represents circumferential shortening in the short-axis plane, its contribution to global systolic function is relatively limited, making it less sensitive than RV GLS and GRS in detecting the responses to pressure unloading.

CTEPH primarily impairs LV function through altering diastolic function [26], consistent with the preserved LV contractility observed before and after BPA in our study. LA conduit strain improved significantly following BPA, while LA total and booster strain remained unchanged, reflecting a preferential recovery of diastolic atrioventricular coupling. As previously shown by Gerges et al [38], RV pressure overload in CTEPH impairs LV relaxation and increases LV diastolic filling pressure; therefore, RV pressure reduction after BPA may lower LV diastolic filling pressure and partially recover LV compliance. This is further supported by the strong correlation between NT-proBNP reduction and improvement in LA conduit strain after BPA, both reflecting alleviation of myocardial wall stress and recovery of LV diastolic function.

Our data regarding RA strain changes after BPA differed from Yamasaki et al [39], who reported improved RA reservoir and conduit functions without recovery of contractile function, but are consistent with Leong et al [8], who observed no significant change in peak RA strain following pulmonary endarterectomy. RA phasic dysfunction likely occurs at an advanced disease stage, reflecting progressive RV diastolic dysfunction and deteriorating ventricular-atrial coupling [40]. In our cohort, preserved RA booster strain at baseline may act as a compensatory mechanism for declining reservoir and conduit functions to maintain RV diastolic filling until decompensation [33]. This discrepancy among studies may therefore relate to heterogeneity in the study population, including different stages of RV maladaptation and comorbidities. Moreover, advanced remodeling may not be readily reversible after relief of pressure overload.

### Study limitations

This prospective single-center study had a relatively small sample size, particularly in the subgroup undergoing follow-up CMR after BPA, which is partly attributable to the rarity of CTEPH and the challenges of implementing a longitudinal design. Larger and multicenter studies are warranted to validate the robustness and generalizability of these findings. Additionally, the exclusion of 7 patients without follow-up CMR may have introduced potential selection bias. The absence of multiplicity adjustment may increase the risk of type I error. The variability in post-BPA follow-up intervals may have potentially affected the findings; a standardized CMR follow-up schedule in the future would bolster consistency. Although healthy controls could not be fully age-matched, age-adjusted analyses did not alter the findings (Supplemental Tables S3–6). Finally, although hs-cTnl decreased after BPA, we did not directly assess myocardial injury or recovery using tissue characterization techniques. Further studies incorporating advanced CMR quantification are warranted to elucidate the myocardial response to chronic pressure overload and the effects of treatment.

## Conclusions

CTEPH is characterized by prominent right heart impairment, including marked chamber dilation and reduced systolic function, accompanied by early biventricular diastolic abnormalities as reflected by impaired biatrial strain, without significant alterations in EAT. Furthermore, BPA could improve right heart structure and function and partially reverse atrial deformation, indicating reversible cardiac remodeling following relief of pressure overload.

## Supplementary information

ELECTRONIC SUPPLEMENTARY MATERIAL

## Data Availability

The datasets used and/or analyzed during the current study are available from the corresponding author on reasonable request.

## Abbreviations

BPA: Balloon pulmonary angioplasty
CI: Cardiac index
CK: Creatine kinase
CK-MB: Creatine kinase muscle-brain fraction
CMR: Cardiac magnetic resonance
CO: Cardiac output
CTEPH: Chronic thromboembolic pulmonary hypertension
EAT: Epicardial adipose tissue
EF: Ejection fraction
ERS: European Respiratory Society
ESC: European Society of Cardiology
FT: Feature-tracking
GCS: Global circumferential strain
GLS: Global longitudinal strain
GRS: Global radial strain
hs-cTnI: High-sensitivity cardiac troponin I
LA: Left atrial
LV: Left ventricular
mPAP: Mean pulmonary arterial pressure
NT-proBNP: N-terminal pro-B-type natriuretic peptide
PH: Pulmonary hypertension
PVR: Pulmonary vascular resistance
RA: Right atrial
RHC: Right heart catheterization
RV: Right ventricular
εa: Booster strain
εe: Conduit strain
εs: Total/reservoir strain

## References

[1] Robbins IM, Pugh ME, Hemnes AR (2017) Update on chronic thromboembolic pulmonary hypertension. Trends Cardiovasc Med 27:29–37 10.1016/j.tcm.2016.05.010 27345156

[2] Humbert M, Kovacs G, Hoeper MM et al (2023) 2022 ESC/ERS Guidelines for the diagnosis and treatment of pulmonary hypertension. Eur Respir J 61:2200879 10.1183/13993003.00879-2022 36028254

[3] Hopman L, Mulder MJ, van der Laan AM et al (2021) Impaired left atrial reservoir and conduit strain in patients with atrial fibrillation and extensive left atrial fibrosis. J Cardiovasc Magn Reson 23:131 10.1186/s12968-021-00820-6 PMC8582184 34758820

[4] Brand A, Frumkin D, Hubscher A et al (2021) Phasic left atrial strain analysis to discriminate cardiac amyloidosis in patients with unclear thick heart pathology. Eur Heart J Cardiovasc Imaging 22:680–687 10.1093/ehjci/jeaa043 32243500

[5] Jain S, Kuriakose D, Edelstein I et al (2019) Right atrial phasic function in heart failure with preserved and reduced ejection fraction. JACC Cardiovasc Imaging 12:1460–1470 10.1016/j.jcmg.2018.08.020 PMC6461533 30343071

[6] Wang Y, Zhao S, Lu M (2024) State-of-the art cardiac magnetic resonance in pulmonary hypertension—an update on diagnosis, risk stratification and treatment. Trends Cardiovasc Med 34:161–171 10.1016/j.tcm.2022.12.005 36574866

[7] Sato T, Tsujino I, Ohira H et al (2015) Right atrial volume and reservoir function are novel independent predictors of clinical worsening in patients with pulmonary hypertension. J Heart Lung Transplant 34:414–423 10.1016/j.healun.2015.01.984 25813768

[8] Leong K, Howard L, Giudice FL et al (2023) Utility of cardiac magnetic resonance feature tracking strain assessment in chronic thromboembolic pulmonary hypertension for prediction of REVEAL 2.0 high risk status. Pulm Circ 13:e12116 10.1002/pul2.12116 PMC9947220 36843875

[9] Kishiki K, Singh A, Narang A et al (2019) Impact of SEvere Pulmonary Arterial Hypertension on the Left Heart and Prognostic Implications. J Am Soc Echocardiogr 32:1128–1137 10.1016/j.echo.2019.05.008 PMC7147873 31278050

[10] Hulten EA, Bradley AJ (2021) Cardiac magnetic resonance evaluation of pulmonary arterial hypertension: Transforming from supplementary to primary imaging modality? JACC Cardiovasc Imaging 14:943–946 10.1016/j.jcmg.2020.11.022 PMC8489254 33454268

[11] Chen Y, Li J, Li F et al (2024) Impact of epicardial adipose tissue on right cardiac function and prognosis in pulmonary arterial hypertension. Chest 165:1211–1223 10.1016/j.chest.2023.11.039 38040053

[12] Lang I, Meyer BC, Ogo T et al (2017) Balloon pulmonary angioplasty in chronic thromboembolic pulmonary hypertension. Eur Respir Rev 26:490–502 10.1183/16000617.0119-2016 PMC9489135 28356406

[13] Roller FC, Schussler A, Hasse A et al (2022) Effects of BPA on right ventricular mechanical dysfunction in patients with inoperable CTEPH—A cardiac magnetic resonance study. Eur J Radiol 147:110111 10.1016/j.ejrad.2021.110111 34952330

[14] Kawakubo M, Yamasaki Y, Kamitani T et al (2019) Clinical usefulness of right ventricular 3D area strain in the assessment of treatment effects of balloon pulmonary angioplasty in chronic thromboembolic pulmonary hypertension: comparison with 2D feature-tracking MRI. Eur Radiol 29:4583–4592 10.1007/s00330-019-6008-3 30790024

[15] Ohno K, Suwa K, Akita K, Sato R, Satoh T, Maekawa Y (2025) Hemodynamics assessment using 4D flow CMR before and after balloon pulmonary angioplasty in chronic thromboembolic pulmonary hypertension: a retrospective observational study. BMC Cardiovasc Disord 25:621 10.1186/s12872-025-05068-x PMC12369117 40841600

[16] Schulz-Menger J, Bluemke DA, Bremerich J et al (2020) Standardized image interpretation and post-processing in cardiovascular magnetic resonance—2020 update: Society for Cardiovascular Magnetic Resonance (SCMR): Board of Trustees Task Force on Standardized Post-Processing. J Cardiovasc Magn Reson 22:19 10.1186/s12968-020-00610-6 PMC7066763 32160925

[17] Lim C, Blaszczyk E, Riazy L et al (2021) Quantification of myocardial strain assessed by cardiovascular magnetic resonance feature tracking in healthy subjects-influence of segmentation and analysis software. Eur Radiol 31:3962–3972 10.1007/s00330-020-07539-5 PMC8128822 33277669

[18] Li L, Chen X, Yin G et al (2020) Early detection of left atrial dysfunction assessed by CMR feature tracking in hypertensive patients. Eur Radiol 30:702–711 10.1007/s00330-019-06397-0 31515621

[19] Cai Q, Zhao Z, Gao J et al (2025) Normal values for atrial deformation measured by feature-tracking cardiac MRI: a meta-analysis. J Magn Reson Imaging 61:882–898 10.1002/jmri.29465 38807354

[20] Li Y, Xu Y, Tang S et al (2022) Left atrial function predicts outcome in dilated cardiomyopathy: fast long-axis strain analysis derived from MRI. Radiology 302:72–81 10.1148/radiol.2021210801 34698565

[21] Leng S, Tan RS, Zhao X, Allen JC, Koh AS, Zhong L (2018) Validation of a rapid semi-automated method to assess left atrial longitudinal phasic strains on cine cardiovascular magnetic resonance imaging. J Cardiovasc Magn Reson 20:71 10.1186/s12968-018-0496-1 PMC6219067 30396356

[22] Kramer CM, Borlaug BA, Zile MR et al (2025) Tirzepatide reduces LV mass and paracardiac adipose tissue in obesity-related heart failure: SUMMIT CMR substudy. J Am Coll Cardiol 85:699–706 10.1016/j.jacc.2024.11.001 39566869

[23] Vonk-Noordegraaf A, Haddad F, Chin KM et al (2013) Right heart adaptation to pulmonary arterial hypertension: physiology and pathobiology. J Am Coll Cardiol 62:D22–D33 10.1016/j.jacc.2013.10.027 24355638

[24] Braams NJ, van Leeuwen JW, Vonk Noordegraaf A et al (2021) Right ventricular adaptation to pressure-overload: Differences between chronic thromboembolic pulmonary hypertension and idiopathic pulmonary arterial hypertension. J Heart Lung Transplant 40:458–466 10.1016/j.healun.2021.02.018 33745783

[25] Vonk Noordegraaf A, Chin KM, Haddad F et al (2019) Pathophysiology of the right ventricle and of the pulmonary circulation in pulmonary hypertension: an update. Eur Respir J 53:1801900 10.1183/13993003.01900-2018 PMC6351344 30545976

[26] Vos JL, Leiner T, van Dijk APJ et al (2022) Cardiovascular magnetic resonance-derived left ventricular intraventricular pressure gradients among patients with precapillary pulmonary hypertension. Eur Heart J Cardiovasc Imaging 24:78–87 10.1093/ehjci/jeab294 34993533

[27] Friedberg MK (2018) Imaging Right-Left Ventricular Interactions. JACC Cardiovasc Imaging 11:755–771 10.1016/j.jcmg.2018.01.028 29747850

[28] Simonneau G, Montani D, Celermajer DS et al (2019) Haemodynamic definitions and updated clinical classification of pulmonary hypertension. Eur Respir J 53:1801913 10.1183/13993003.01913-2018 PMC6351336 30545968

[29] Waziri F, Ringgaard S, Mellemkjaer S et al (2020) Long-term changes of right ventricular myocardial deformation and remodeling studied by cardiac magnetic resonance imaging in patients with chronic thromboembolic pulmonary hypertension following pulmonary thromboendarterectomy. Int J Cardiol 300:282–288 10.1016/j.ijcard.2019.09.038 31744721

[30] Wang Y, Guo D, Liu M et al (2022) Assessment of right ventricular remodeling in chronic thromboembolic pulmonary hypertension by 2D-speckle tracking echocardiography: a comparison with cardiac magnetic resonance. Front Cardiovasc Med 9:999389 10.3389/fcvm.2022.999389 PMC9712438 36465465

[31] Mottram PM, Marwick TH (2005) Assessment of diastolic function: what the general cardiologist needs to know. Heart 91:681–695 10.1136/hrt.2003.029413 PMC1768877 15831663

[32] Cameli M, Mandoli GE, Loiacono F, Dini FL, Henein M, Mondillo S (2016) Left atrial strain: a new parameter for assessment of left ventricular filling pressure. Heart Fail Rev 21:65–76 10.1007/s10741-015-9520-9 26687372

[33] Querejeta Roca G, Campbell P, Claggett B, Solomon SD, Shah AM (2015) Right atrial function in pulmonary arterial hypertension. Circ Cardiovasc Imaging 8:e003521 10.1161/CIRCIMAGING.115.003521 PMC4629509 26514759

[34] Yan WF, Wang J, Li Y et al (2025) Epicardial adipose tissue volume is associated with impaired left ventricular strain in patients with metabolic syndrome: the mediating role of myocardial energetic efficiency. Cardiovasc Diabetol 24:325 10.1186/s12933-025-02859-6 PMC12335042 40783739

[35] Li W, Yang T, Quan RL et al (2021) Balloon pulmonary angioplasty reverse right ventricular remodelling and dysfunction in patients with inoperable chronic thromboembolic pulmonary hypertension: a systematic review and meta-analysis. Eur Radiol 31:3898–3908 10.1007/s00330-020-07481-6 33201287

[36] Leather HA, Ama R, Missant C, Rex S, Rademakers FE, Wouters PF (2006) Longitudinal but not circumferential deformation reflects global contractile function in the right ventricle with open pericardium. Am J Physiol Heart Circ Physiol 290:H2369–H2375 10.1152/ajpheart.01211.2004 16399859

[37] Suever JD, Wehner GJ, Jing L et al (2017) Right ventricular strain, torsion, and dyssynchrony in healthy subjects using 3D spiral cine DENSE magnetic resonance imaging. IEEE Trans Med Imaging 36:1076–1085 10.1109/TMI.2016.2646321 PMC5711416 28055859

[38] Gerges C, Pistritto AM, Gerges M et al (2023) Left ventricular filling pressure in chronic thromboembolic pulmonary hypertension. J Am Coll Cardiol 81:653–664 10.1016/j.jacc.2022.11.049 36792280

[39] Yamasaki Y, Abe K, Kamitani T et al (2020) Balloon pulmonary angioplasty improves right atrial reservoir and conduit functions in chronic thromboembolic pulmonary hypertension. Eur Heart J Cardiovasc Imaging 21:855–862 10.1093/ehjci/jeaa064 32359071

[40] Vonk Noordegraaf A, Westerhof BE, Westerhof N (2017) The relationship between the right ventricle and its load in pulmonary hypertension. J Am Coll Cardiol 69:236–243 10.1016/j.jacc.2016.10.047 28081831

