# Supplementary material for: Assessment of atrioventricular interaction and response to balloon pulmonary angioplasty in chronic thromboembolic pulmonary hypertension using cardiac MRI
Source: Insights Imaging. 2026 Nov 12;17:291. doi: 10.1186/s13244-026-02453-8 (PMC13759951; doi:10.1186/s13244-026-02453-8)
Supplement: Supplementary file 1 [file 13244_2026_2453_MOESM1_ESM.pdf]

Assessment of Atrioventricular Interaction and Response to Balloon Pulmonary Angioplasty in Chronic Thromboembolic Pulmonary Hypertension Using Cardiac MRI

ELECTRONIC SUPPLEMENTARY MATERIAL

Table of Contents

Supplemental Methods ………………………………………………………………………………. .2

Supplemental Figure S1 ……………………………………………………………………………..6

Supplemental Figure S2 ……………………………………………………………………………..7

Supplemental Table S1 ……………………………………………………………………………8

Supplemental Table S2 ……………………………………………………………………………9

Supplemental Table S3 …………………………………………………………………………10

Supplemental Table S4 …………………………………………………………………………11

Supplemental Table S5 …………………………………………………………………………12

Supplemental Table S6 …………………………………………………………………………13

CTEPH definition and exclusion criteria

According to the updated 2022 ESC/ERS Guidelines [2], CTEPH was defined as a mean pulmonary arterial pressure (mPAP) >20 mm Hg, pulmonary arterial wedge pressure (PAWP) ≤15 mm Hg, and PVR >2 Wood units (WU), confirmed by right heart catheterization (RHC) examination after at least 3 months of effective anticoagulation.

Main exclusion criteria for CTEPH patients were: (1) age <18 years, absence of PH confirmed by RHC, or incomplete clinical data; (2) concomitant other PH subtypes (*e.g*. PH due to left ventricular (LV) disease, lung disease or unclear etiologies); (3) mixed phenotypes, including LV hypertrophy or hypertensive heart disease; LV ejection (EF) <50% or a history of heart failure; (4) claustrophobia; (5) presence of metallic objects or implanted medical devices in the body; (6) myocardial infarction within 6 months prior to BPA, persistent atrial fibrillation or severe arrythmia at enrollment; (7) severe hepatic dysfunction or type 1 diabetes mellitus; and (8) conditions precluding adequate CMR image quality or post-processing analysis (*e.g.*, inability to cooperate with breath-holding or significant motion artifacts).

CMR protocol - imaging acquisition

Imaging acquisition parameters were as follows: for the LV long-axis (LAX) view, repetition time/echo time (TR/TE) =2.8/1.4 ms or 2.7/1.4 ms, flip angle (FA) =45°, bandwidth =125 kHz, field of view (FOV) =320 × 320 mm^2, matrix =256 × 256, slice thickness =8 mm, interslice gap =0 mm, and number of excitations (NEX) =1. For the 4-chamber view, TR/TE =3.0/1.5 ms, FA =45°, bandwidth =125 kHz, FOV =350 × 350 mm^2, matrix =320 × 320, slice thickness =8 mm, interslice gap =0 mm, and NEX =1. For the short-axis (SAX) cine stacks, TR/TE =2.8/1.4 ms or 2.5/1.3 ms, FA =45°, bandwidth =125 kHz, FOV =353 × 353 mm^2, matrix =320 × 320, slice thickness =8 mm, interslice gap =1 mm, and NEX =1; 8-14 slices were obtained from base to apex.

Detailed CMR feature-tracking analysis

In the tissue-tracking module, LV endocardial and epicardial contours were semi-automatically delineated at end-diastole on SAX and LAX (2-, 3-, and 4-chamber) cine images (**Figure 2a**). RV endocardial contours were also semi-automatically delineated, whereas RV epicardial contours were manually traced. Global longitudinal strain (GLS) was assessed from the three LAX views, while global circumferential (GCS) and radial strain (GRS) were derived from three SAX slices (basal, midventricular, and apical) in all subjects [17]. Endocardial and epicardial contours were automatically propagated by the software throughout the cardiac cycle (**Figure 2b**).

LA FT-derived longitudinal strain was obtained from 2- and 4-chamber cine views, as previously described [18-20]. Briefly, LA endocardial and epicardial borders were manually traced on both 2- and 4-chamber cine images using the phase corresponding to the maximal LA volume, following atrial contraction (**Figure 2c**). Pulmonary veins and the LA appendage were excluded from the endocardial contour. The software then automatically propagated the delineated borders throughout the entire cardiac cycle. All LA strain metrics were averaged between 2- and 4-chamber values.

Figure 2d illustrates the measurement of RA longitudinal strain. RA endocardial and epicardial contours were manually delineated in the 4-chamber view using a point-and-click approach at the phase of maximal RA volume [5]. The superior vena cava and RA appendage were also excluded from the endocardial border. The borders were then automatically tracked throughout the cardiac cycle, and the tracking accuracy was visually and manually adjusted when necessary.

Atrial longitudinal strain (ε) at any time point (*t*) during the cardiac cycle was calculated using the following formula:

$$\varepsilon_{(t)} = \left[\frac{\left(L_{(t)} - L_0\right)}{L_0}\right] \times 100$$

Where $L_{(t)}$ represents the atrial length at time *t,* and L_0 denotes the length at the time of minimal atrial volume (LV end-diastolic phase) [5, 21]. Phasic LA and RA longitudinal strain components, including total (reservoir) strain (εs), conduit strain (εe), and booster strain (εa) were subsequently derived [5]. Further illustration is provided in **Supplemental Figure S1.**

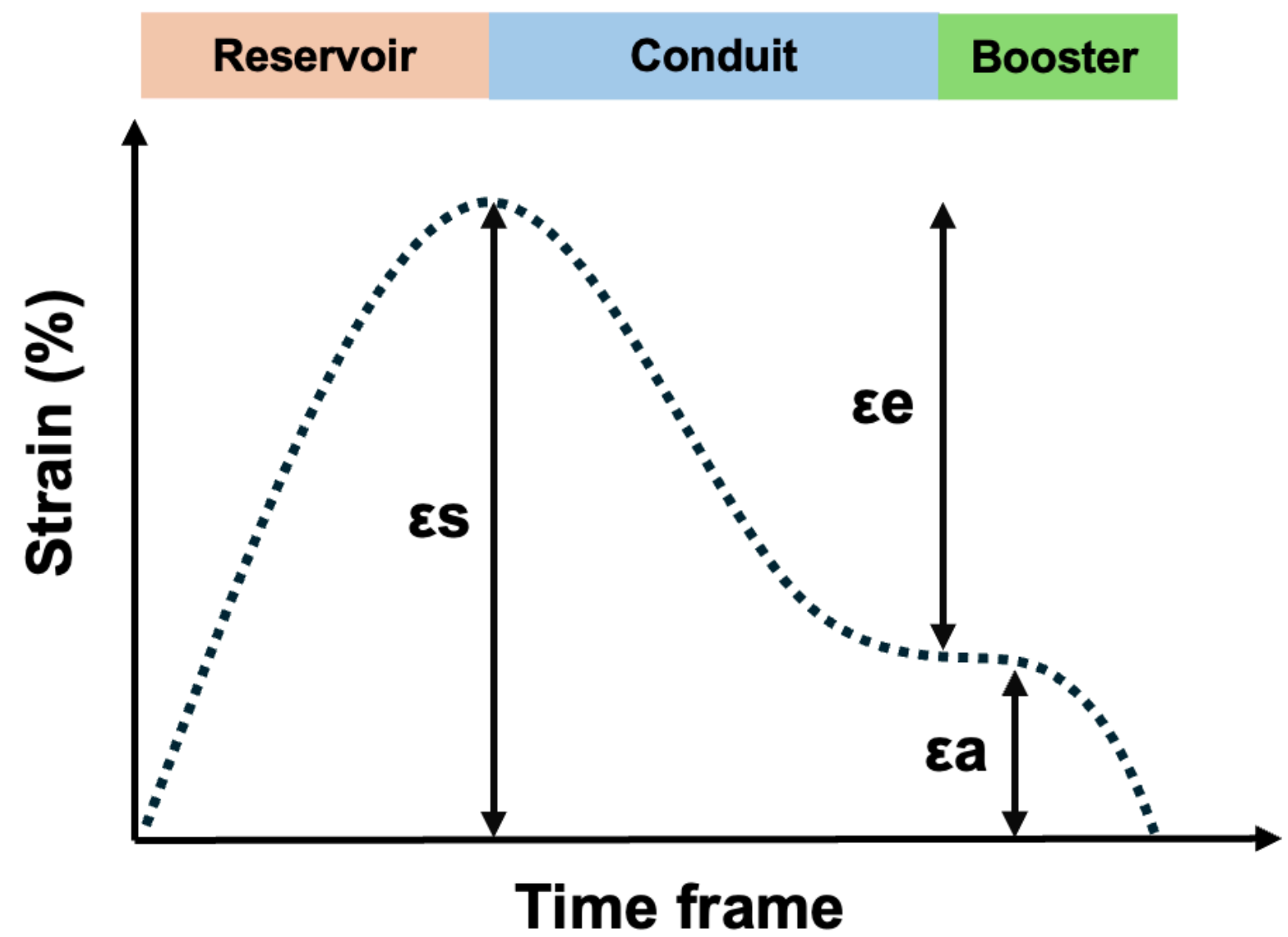

Supplemental Figure S1 Example of phasic functional curve for quantification of atrial strain.

Atrial strain represents three primary phasic functions: (1) reservoir function, where atrial total (reservoir) strain (εs) reflects the collection of systemic venous return during ventricular systole; (2) conduit function, where conduit strain (εe) represents passive blood filling into the ventricle during early-mid diastole; (3) contractile (booster pump) function, where booster strain (εa) corresponds to active atrial contraction in late ventricular diastole. εs =total strain; εe =conduit strain; εa =booster strain.

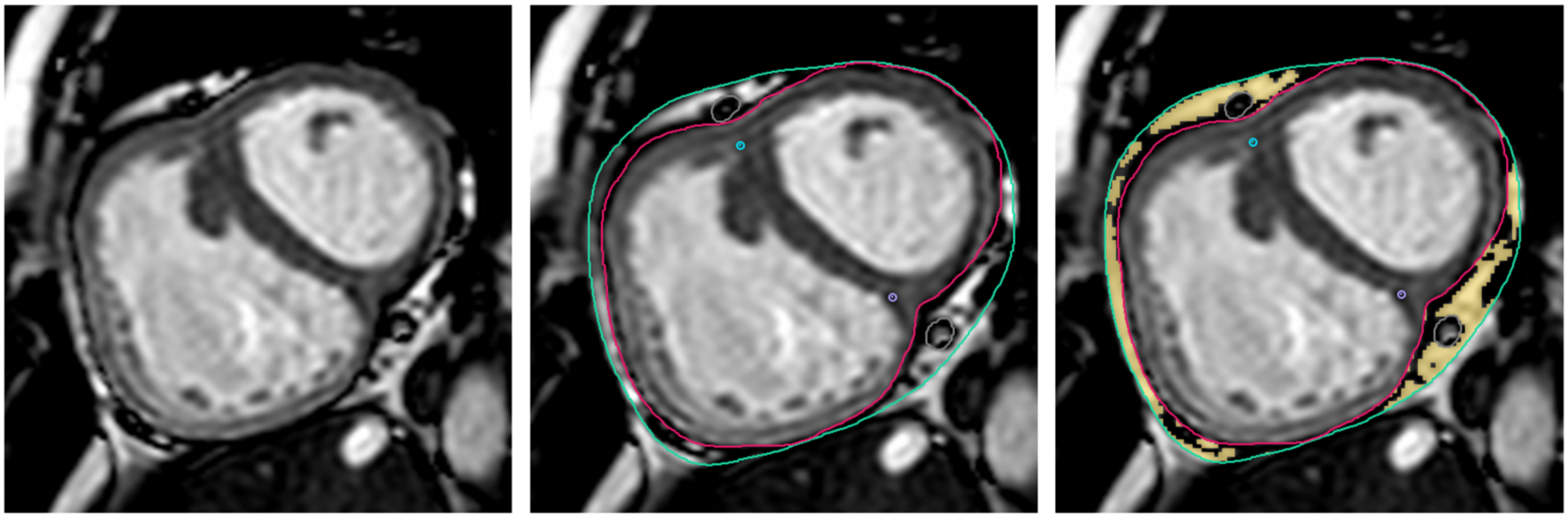

Supplemental Figure S2 Assessment of the epicardial adipose tissue on cine images. Representative near-basal short-axis ventricular cine slice is shown (left). The middle plot shows the manual delineation of the epicardium (red) and visceral pericardium (green), with intravascular lumina within the traced region manually excluded (grey). The yellow mask from base to apex at end-diastole quantified as epicardial adipose tissue (EAT) volume or mass (right).

Supplemental Table S1 Baseline demographic and clinical characteristics of follow-up CTEPH patients with and without follow-up CMR

	**Without follow-up CMR** (n =7)	**With follow-up CMR** (n =25)	***p*-value**
Demographics			
Age, years	59 ±7	60 ±12	0.791
Female, n (%)	4 (57.1)	19 (76)	0.370
Height, cm	164 ±8	159 ±8	0.165
Weight, kg	67 ±10	60 ±8	0.138
Body surface area, m²	1.7 ±0.2	1.6 ±0.1	0.101
BMI, kg/m²	25 ±3	24 ±3	0.464
Clinical parameters			
6MWD, m	441 ±35	390 ±92	0.115
WHO class, n (%)			-
I	-	1 (4.2)	
II	3 (42.9)	14 (58.3)	
III	-	8 (33.3)	
IV	-	1 (4.2)	
Blood biomarkers			
NT-proBNP, pg/mL	108 (90, 241)	594 (157, 1056)	**0.020**
hs-cTnI, pg/mL	2.7 (2, 5)	7.8 (5.2, 10.5)	**0.002**
CK, U/L	88 ±44	78 ±38	0.581
CK-MB, U/L	9 (7, 13)	10 (9, 12.8)	0.352
RHC metrics			
mPAP, mmHg	33 ±11	44 ±11	**0.048**
SVCP, mmHg	6 ±2	6 ±3	0.773
IVCP, mmHg	7 ±2	6 ±3	0.355
RAP, mmHg	6 ±2	6 ±3	0.853
PAWP, mmHg	9 ±3	9 ±3	0.968
PVR, WU	5 ±3	8 ±4	**0.040**
CO†, L/min	5.9 ±1.5	5.0 ±2.0	0.164
CI†, L/min/m²	3.4 ±0.9	3.2 ±1.4	0.398
PH severity‡, n (%)			**0.023**
Mild	4 (57.1)	3 (12)	
Moderate	3 (42.9)	14 (56)	
Severe	0 (0)	8 (32)	

Values are presented as mean ±SD or median (Q1, Q3) for continuous, and n (%) for categorical data. Significant p-values are shown in ***bold****. WHO class was not compared due to missing data in 4 patients without follow-up CMR.*

† CO and CI were measured by Fick's method.

‡ PH severity was defined as mild (20 mmHg < mPAP < 30 mmHg), moderate (30 mmHg ≤ mPAP < 50 mmHg), and severe (mPAP ≥ 50 mmHg) by 2022 ESC/ERS Guidelines [2].

Abbreviations: 6MWD =6-minute walk distance; BMI =body mass index; CI =cardiac index; CMR =cardiac magnetic resonance; CO =cardiac output; CK-MB =creatine kinase muscle-brain fraction; CTEPH =chronic thromboembolic pulmonary hypertension; hs-cTnI =high-sensitivity cardiac troponin I; IVCP =inferior vena cava pressure; mPAP =mean pulmonary arterial pressure; NT-proBNP =N-terminal pro-B-type natriuretic peptide; PAWP =pulmonary artery wedge pressure; PH =pulmonary hypertension; PVR =pulmonary vascular resistance; RAP =right atrial pressure; RHC =right heart catheterization; SVCP =superior vena cava pressure; WU =Wood units.

**Supplemental Table S2** Intra- and inter-observer reproducibility of strain and EAT measurements				
	**Intra-observer ICC**	**95%CI**	**Inter-observer ICC**	**95%CI**
LV GLS	0.952	0.862–0.983	0.947	0.850–0.982
LV GCS	0.947	0.851–0.982	0.903	0.737–0.966
LV GRS	0.914	0.763–0.970	0.913	0.762–0.970
RV GLS	0.940	0.832–0.980	0.938	0.827–0.979
RV GCS	0.914	0.763–0.970	0.905	0.742–0.967
RV GRS	0.898	0.723–0.964	0.897	0.722–0.964
LA total strain (εs)	0.938	0.827–0.979	0.932	0.809–0.977
LA conduit strain (εe)	0.937	0.822–0.978	0.955	0.873–0.985
LA booster strain (εa)	0.898	0.724–0.965	0.879	0.678–0.958
RA total strain (εs)	0.920	0.780–0.972	0.916	0.769–0.971
RA conduit strain (εe)	0.858	0.629–0.950	0.902	0.735–0.966
RA booster strain (εa)	0.911	0.757–0.969	0.898	0.724–0.964
Total EAT volume index	0.932	0.810–0.977	0.929	0.803–0.976

Intra- and inter-observer reproducibility analyses were performed in 15 (28.8%) randomly selected patients.

Abbreviations: CI =confidence interval; EAT =epicardial adipose tissue; GCS =global circumferential strain; GLS =global longitudinal strain; GRS =global radial strain; ICC =intraclass correlation coefficient; LA =left atrial; LV =left ventricular; RA =right atrial; RV =right ventricular.

Supplemental Table S3 Multivariable linear regression analysis of clinical factors associated with LA strain in CTEPH patients (n =52)

**Variables**	**LA total strain**		**LA booster strain**		**LA conduit strain**	
Demographics	β^a	β^b	β^a	β^b	β^a	β^b
Age	-0.09091		0.1221		-0.2167	
Gender	-7.195	-7.578	-3.307	-2.722	-5.188	-6.227
Height	0.3674	0.4172	0.09882	0.03564	0.2443	0.3564
Weight	-0.2064	-0.2191	0.007053	0.02551	-0.1970	-0.2297
Hemodynamics						
mPAP	-0.4451	-0.4172	-0.3766	-0.4548	0.05332	0.1921
PAWP	-0.3426	-0.4073	-0.09728	0.01992	-0.3148	-0.5228
PVR	-0.3513	-0.4237	0.5784	0.7957	-1.163	-1.549
Blood biomarkers						
NT-proBNP	-0.002515	-0.002648	-0.001167	-0.001049	-0.0009072	-0.001117
hs-cTnI	-0.2629	-0.2668	-0.1093	-0.1031	-0.1488	-0.1598
R^2	0.4858	0.4809	0.4381	0.4044	0.4729	0.4079

*β^a represents the standardized regression coefficient, and β^b represents the age-adjusted coefficient from the multivariable linear regression analysis. Variables included in the multivariable linear model are indicated in **bold**. *p <0.05, **p <0.01.*

Abbreviations: CTEPH =chronic thromboembolic pulmonary hypertension; hs-cTnI =high-sensitivity cardiac troponin I; LA =left atrial; mPAP =mean pulmonary arterial pressure; NT-proBNP =N-terminal pro-B-type natriuretic peptide; PAWP =pulmonary artery wedge pressure; PVR =pulmonary vascular resistance.

Supplemental Table S4 Multivariable linear regression analysis of clinical factors associated with RA strain in CTEPH patients (n =52)

**Variables**	**RA total strain**		**RA booster strain**		**RA conduit strain**	
Demographics	$β^a$	$β^b$	$β^a$	$β^b$	$β^a$	$β^b$
Age	-0.1166		-0.005744		-0.1108	
Gender	**-10.66***	**-11.15***	-3.079	-3.104	**-7.580***	**-8.048***
Height	0.1218	0.1857	-0.3343	-0.3312	**0.4561***	**0.5169***
Weight	-0.08152	-0.09787	0.01194	0.01113	-0.09346	-0.1090
Hemodynamics						
mPAP	**-0.7597***	**-0.7240***	**-0.6036***	**-0.6018***	-0.1561	-0.1222
PAWP	1.288	1.205	0.8907	0.8866	0.3971	0.3182
PVR	1.198	1.105	**1.740***	**1.736***	-0.5424	-0.6307
Blood biomarkers						
NT-proBNP	-0.003414	-0.003584	-0.002504	-0.002513	-0.0009099	-0.001072
hs-cTnI	-0.01226	-0.01730	0.1594	0.1591	-0.1716	-0.1764
R^2	0.5772	0.5652	0.4349	0.4348	0.6011	0.5757

*$β^a$ represents the standardized regression coefficient, and $β^b$ represents the age-adjusted coefficient from the multivariable linear regression analysis. Variables included in the multivariable linear model are indicated in **bold**. *p <0.05, **p <0.01.*

Abbreviations: CTEPH =chronic thromboembolic pulmonary hypertension; hs-cTnI =high-sensitivity cardiac troponin I; mPAP =mean pulmonary arterial pressure; NT-proBNP =N-terminal pro-B-type natriuretic peptide; PAWP =pulmonary artery wedge pressure; PVR =pulmonary vascular resistance; RA =right atrial.

Supplemental Table S5 Multivariable linear regression analysis of clinical factors associated with LV strain in CTEPH patients (n =52)

**Variables**	**LV GLS**		**LV GCS**		**LV GRS**	
Demographics	β^a	β^b	β^a	β^b	β^a	β^b
Age	-0.0138		0.01827		-0.139	
Gender	**5.022****	**4.964****	2.852	2.929	1.367	0.7808
Height	-0.1827	-0.1752	-0.1183	-0.1283	-0.4604	-0.3842
Weight	0.1109	0.109	0.04068	0.04324	0.2789	0.2594
Hemodynamics						
mPAP	0.0694	0.07363	-0.03797	-0.04357	0.3129	0.3555
PAWP	-0.3889	-0.3987	-0.08865	-0.07564	-0.7748	-0.8738
PVR	0.197	0.186	0.3669	0.3815	-0.8867	-9974
Blood biomarkers						
NT-proBNP	-0.00031	-0.00033	-0.001034	-0.001007	0.004002	0.003799
hs-cTnI	-0.01397	-0.01456	0.07165	0.07244	-0.3943	-0.4003
R^2	0.4181	0.4165	0.2868	0.2818	0.1298	0.1147

*β^a represents the standardized regression coefficient, and β^b represents the age-adjusted coefficient from the multivariable linear regression analysis. Variables included in the multivariable linear model are indicated in **bold**. *p <0.05, **p <0.01.*

Abbreviations: CTEPH =chronic thromboembolic pulmonary hypertension; GCS =global circumferential strain; GLS =global longitudinal strain; GRS =global radial strain; hs-cTnI =high-sensitivity cardiac troponin I; LV =left ventricular; mPAP =mean pulmonary arterial pressure; NT-proBNP =N-terminal pro-B-type natriuretic peptide; PAWP =pulmonary artery wedge pressure; PVR =pulmonary vascular resistance.

Supplemental Table S6 Multivariable linear regression analysis of clinical factors associated with RV strain in CTEPH patients (n =52)

**Variables**	**RV GLS**		**RV GCS**		**RV GRS**	
Demographics	β^a	β^b	β^a	β^b	β^a	β^b
Age	0.09548		0.04337		-0.07682	
Gender	4.493	4.895	1.727	1.91	-4.014	-4.338
Height	-0.3134	-0.3657	-0.1861	-0.2099	**0.4379***	**0.48***
Weight	**0.3127****	**0.3261****	-0.02208	-0.01599	0.01227	0.001499
Hemodynamics						
mPAP	0.08931	0.06005	0.0663	0.05301	-0.1578	-0.1342
PAWP	-0.5358	-0.4679	**-0.5681***	**-0.5372***	**1.068***	**1.013***
PVR	0.1012	0.1772	-0.1529	-0.1183	0.3081	0.2469
Blood biomarkers						
NT-proBNP	0.002962	-0.003102	**0.002228****	**0.002292****	**-0.003299***	**-0.003411***
hs-cTnI	-0.1266	-0.1225	-0.07301	-0.07113	0.04509	0.04177
R^2	0.3755	0.3531	0.4282	0.4103	0.4212	0.4053

*β^a represents the standardized regression coefficient, and β^b represents the age-adjusted coefficient from the multivariable linear regression analysis. Variables included in the multivariable linear model are indicated in **bold**. *p <0.05, **p <0.01.*

Abbreviations: CTEPH =chronic thromboembolic pulmonary hypertension; GCS =global circumferential strain; GLS =global longitudinal strain; GRS =global radial strain; hs-cTnI =high-sensitivity cardiac troponin I; mPAP =mean pulmonary arterial pressure; NT-proBNP =N-terminal pro-B-type natriuretic peptide; PAWP =pulmonary artery wedge pressure; PVR =pulmonary vascular resistance; RV =right ventricular.
